# Transfer of Pollutants from *Macrocystis pyrifera* to *Tetrapygus niger* in a Highly Impacted Coastal Zone of Chile

**DOI:** 10.3390/toxics9100244

**Published:** 2021-10-01

**Authors:** Nicolás Latorre-Padilla, Andrés Meynard, Jorge Rivas, Loretto Contreras-Porcia

**Affiliations:** 1 Departamento de Ecología y Biodiversidad, Facultad de Ciencias de la Vida, Universidad Andres Bello, Santiago 8370251, Chile; nlatorrepadilla@gmail.com (N.L.-P.); meynardster@gmail.com (A.M.); jrivasperez@gmail.com (J.R.); 2 Centro de Investigación Marina Quintay (CIMARQ), Facultad de Ciencias de la Vida, Universidad Andres Bello, Valparaíso, Quintay 2531015, Chile; 3 Center of Applied Ecology and Sustainability (CAPES), Santiago 8331150, Chile; 4 Instituto Milenio en Socio-Ecología Costera (SECOS), Santiago 8370251, Chile; 5 Programa de Doctorado en Medicina de la Conservación, Facultad de Ciencias de la Vida, Universidad Andres Bello, Santiago 8370251, Chile

**Keywords:** marine contamination, ecosystem health, kelps, sea urchin, heavy metals, PAHs

## Abstract

PAHs and heavy metals are characteristic pollutants in urbanized coastal areas, especially those with industrial activity. Given this context and the ability of *Macrocystis pyrifera* to drift when detached and provide trophic subsidy in coastal systems, we analyzed the potential transfer of pollutants to the herbivore *Tetrapygus niger*, through diet, in an industrialized coastal zone in Central Chile (Caleta Horcón) and characterized the impacted zone using diverse polluted ecotoxicological indices. For this purpose, a culture experiment was conducted where *M. pyrifera* individuals from Algarrobo (control site) were cultivated in Caleta Horcón and then used as food for *T. niger*. The contents of both PAHs and heavy metal contents were subsequently determined in algal tissue and sea urchin gonads as well as in the seawater. The results show that algae cultivated in Caleta Horcón had higher concentrations of naphthalene (NAF) compared to those from a low industrial impact zone (Algarrobo) (2.5 and 1.8 mg kg^−1^, respectively). The concentrations of Cu, As, and Cd were higher in Caleta Horcón than in Algarrobo in both *M. pyrifera* and *T. niger*. For all metals, including Pb, higher concentrations were present in *T. niger* than in *M. pyrifera* (between 5 and 798 times higher). Additionally, as indicated by the toxicological indices *MPI* (0.00804) and *PLI* (10.89), Caleta Horcón is highly contaminated with metals compared to Algarrobo (0.0006 and 0.015, respectively). Finally, the bioconcentration factor (*BCF*) and trophic transfer factor (*TTF*) values were greater than one in most cases, with values in Caleta Horcón exceeding those in Algarrobo by one or two orders of magnitude. This study provides evidence that Caleta Horcón is a highly impacted zone (HIZ) compared to Algarrobo, in addition to evidence that the biomagnification of certain pollutants, including the possible responses to contaminants, are apparently not exclusively transferred to *T. niger* through diet.

## 1. Introduction

Polycyclic aromatic hydrocarbons (PAHs) form one of the most common groups of pollutants in the environment [1,2]. PAHs are organic molecules composed of H and C with two or more aromatic rings, with naphthalene (NAF) being the simplest in structure with only two aromatic rings. The structure of these compounds can provide thermal and photoresistance, making them highly resistant to degradation under natural conditions in the environment, although they may be biodegraded by organisms in small quantities [3,4]. Moreover, they have low water solubility and vapour pressure, which means they can be extremely volatile depending on the altitude and environmental temperature at which they are found [5]. These pollutants mainly originate from fossil fuel extraction, combustion, and spills, as well as the generally incomplete combustion of organic matter, such as wood in forest fires. They are widely distributed at the global level [6,7], and one of the most predominant dispersion mechanisms is transport through air currents upon integration into the atmosphere [6]. PAHs are closely related to human activity and, in marine systems, are present at higher concentrations in urbanized coastal zones, both in the water column and in sediments. Their presence in the environment has generated widespread concern and has been shown to negatively affect both human and environmental health [8,9,10,11].

Another important group of pollutants is heavy metals (HMs) which, according to their ecotoxicological definition, are metallic chemicals with a high density and are toxic at relatively low concentrations, including Cu, As, Cd, and Pb, amongst others [2]. HMs are a natural part of the ecosystem and, specifically in marine habitats, can originate from the atmosphere as particulate matter or from the outflow of rivers [12]. Human activities inevitably increase the incorporation of these pollutants into marine systems [13]. Cu, Zn, and Fe are essential metals, which means that they are necessary for life, but they may be toxic and even lethal at elevated concentrations. These pollutants may negatively affect the organisms by decreasing successful reproduction or even causing death [14]. The bioavailability of HMs is influenced by environmental factors such as temperature, pH, salinity, solubility, and, in some cases, the presence of (and interaction with) other HMs [15,16,17]. Their toxicity is primarily related to the induction and generation of reactive oxygen species (ROS) through the Haber–Weiss reaction, which can cause damage to cellular components, such as DNA, lipids, proteins, and organelles [18,19,20,21]. Similar to PAHs, HMs may have negative health impacts in humans, animals, and ecosystems [14,22].

**Figure 1:**
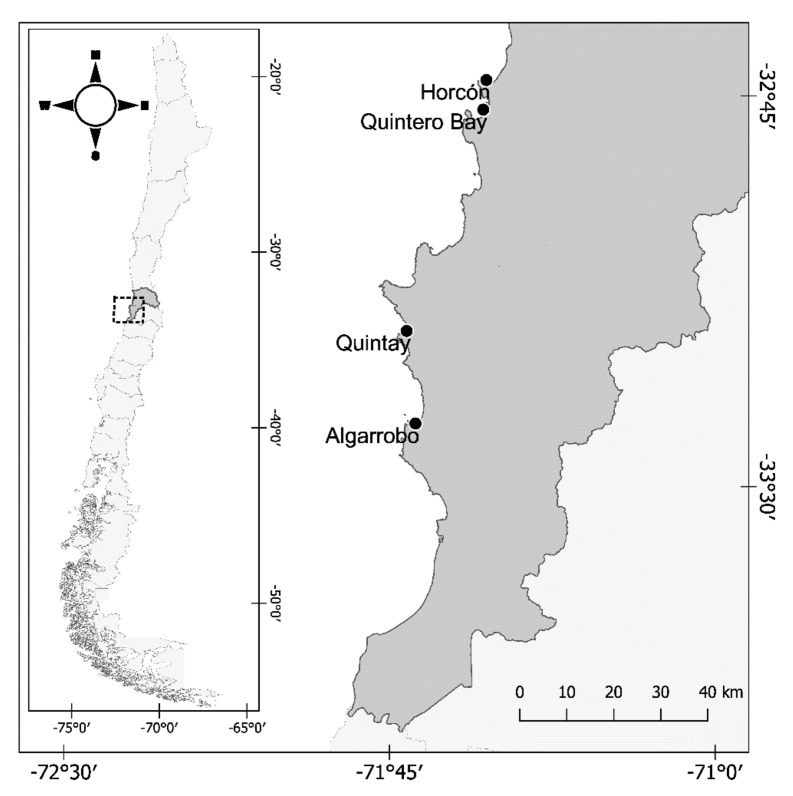
Map of the researched areas in this study. The gray area represents the Valparaíso region of Chile.

Various industrial parks have been installed on the Chilean coast for a variety of purposes, such as mining refineries, energy production, amongst others, including fossil fuel unloading in ports. Specifically in the Valparaíso region, in 1961, the Ventanas industrial park was inaugurated in Quintero Bay (32°44′ S 71°30′ W; Figure 1) where, to date, the common forms of pollution from different operating companies contain significant pollutants, such as PAHs and HMs, which can be absorbed by different organisms, constituting a threat to marine and terrestrial ecosystems [23,24,25,26]. A study recently conducted in this area reported high concentrations of heavy metals in the water column near Quintero Bay: Cu at 28–741 µg L^−1^, As at 9–348 µg L^−1^, Cd at 0.091–0.243 µg L^−1^, and Pb at 0.093–3.425 µg L^−1^; similar concentrations were reported for Caleta Horcón and Cachagua, two sites 5 and 19 km north, respectively, of the industrial park [23]. No recent studies have been performed in relation to the PAH concentrations in Quintero Bay, but frequent oil spills have been reported in this area [24]. Nonetheless, a 2016 report on the pollution status of sites located near the highly industrialized area of Quintero Bay have described the presence of several PAHs, with a relatively high concentration of total PAHs in the seawater (6.23–17.61 µg L^−1^ [25]). 

Pollutants in the environment can accumulate in organisms through direct or indirect routes, the latter primarily occurring through the consumption of organisms at lower trophic levels and biomagnification through the food chain [27,28,29]. PAHs are primarily found in sediments because of their physical and chemical nature, but they may be removed from the seabed and remain bioavailable in the water column due to abiotic factors, such as changes in water flow patterns and temperature [30]. In addition, heavy metals can be dissolved in the water column depending on speciation and their hydrogeochemistry cycles and can be precipitated in the sediment with a high level of bioavailability [13,31]. As PAHs are fat-soluble molecules with a low biodegradation rate, their concentrations inside organisms are higher than those found in the environment. Even when the concentrations of PAHs in the environment are low, their capacity to concentrate in organisms is high. This bioconcentration in aquatic organisms is thermodynamically favored for biodegradable molecules such as PAHs due to the hydrophobicity of these molecules, so the excess will tend toward the lipids of organisms in the water, and this tendency increases with the increase in the molar mass of the PAHs [32].

As mentioned above, PAHs and HMs can become concentrated in organisms, and this process can occur through different routes. For example, in aquatic organisms, these toxicants may enter by diffusion through the gills, direct absorption through the skin, and the ingestion of sediment, detritus, or food [33,34]. Because of the potential transfer of these pollutants through diet, their presence and concentration in the most basal trophic levels are important for understanding the dynamics of PAHs and HMs in food webs. Previous studies have demonstrated that several macroalgal species can bioconcentrate both PAHs and HMs in different ranges of concentration [35,36,37,38]. 

The brown alga *Macrocystis pyrifera*, which is distributed along the Chilean coasts [39,40], provide shelter, food, and substrate for settlement for its associated flora and fauna, and for this reason, sites where it is found are especially rich in chordates, invertebrates, and other algae [41]. This species has the capacity to bioconcentrate HMs, as shown by [37]. Their work provides evidence that a high concentration of Cu and Zn in the water column or culture medium is positively correlated with a high concentration of these two metals in the tissues of *M. pyrifera.* Little information is available in the current literature on the capacity of PAHs to become bioconcentrated in this species. However, several deleterious effects in terms of sporulation, settlement, and germination were recently identified in this kelp and in *Lessonia spicata* exposed to Cu, Cd, and PAHs, as well as their binary and tertiary mixtures [16,17,42]. Conversely, it has been documented that individual *M. pyrifera* detached from the substrate can be passively dispersed over long distances, transporting both adhered animal species and algae; these detached algae are able to survive during drifting and develop reproductive structures [43,44]. At the ecological level, based on these findings and species characteristics, *M. pyrifera* can provide food for both land and aquatic species, such as the sea urchin *Tetrapygus niger* (Molina, 1782), which primarily feeds on kelps washed ashore [45,46]. *T. niger* is considered an ecosystem engineer, structuring the subtidal community and changing the landscape from an erect kelp forest to a coralline barren [47]. Notably, in this species of sea urchin, both sexes present mature gonads throughout the year [48]; therefore, due to the constant development of gametes, pollutants tend to accumulate in the gonads [49]. In this context, the goal of this study was to provide evidence of the transfer of pollutants to *T. niger* from the water column by means of *M. pyrifera* as an intermediary in the food web and to characterize the industrialized coastal zone by means of diverse pollution ecotoxicological indices. 

## 2. Materials and Methods

Fifteen whole individuals of *M. pyrifera*, measuring approximately 4–5 m long, were collected by hand in the Valparaíso Region at Algarrobo beach (33°21′22″ S, 71°39′36″ W), which was defined in this study as a low industrial impact zone (non-impacted zone, NIZ). These individuals were transported in an isolation box and kept wet with sponges at the Benthonic Resource Extraction and Management Area (AMERB) located at the highly impacted zone (HIZ) Caleta Horcón (32°42′24″ S, 71°29′34″ W) (Figure 1). 

**Figure 2:**
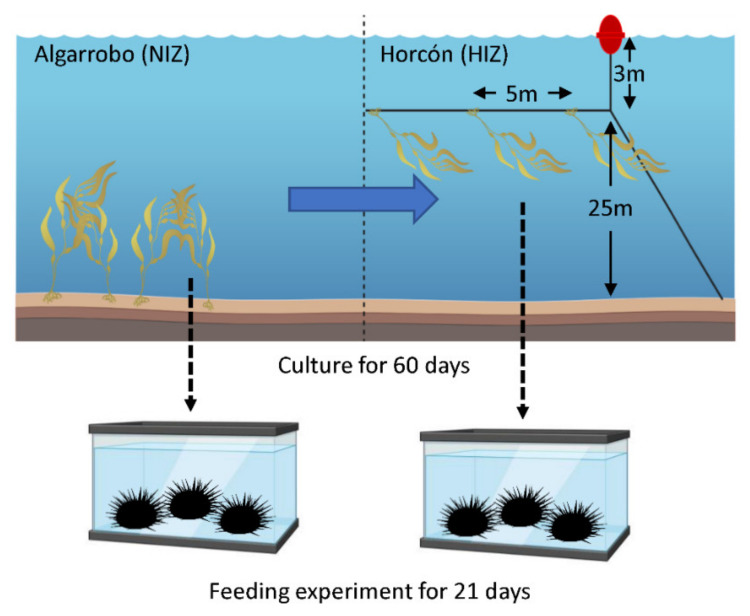
Simplified scheme of the culture and feeding experiments. The blue arrow indicates the origin and destination of the 15 *Macrocystis pyrifera* individuals, and the dashed arrows correspond to the origin of the kelp tissue for the feeding experiment according to the treatments.

This first group of 15 kelps from Algarrobo were cultured in a long-line system previously installed at the HIZ site located within the AMERB under the FIC Algas project funded by GORE Valparaíso (30397482-0) (Figure 2). The installation was performed with local fishers at a depth of 3–5 m, in a line of 75 m, and each kelp was separated by 5 m. The kelps were anchored with reusable tie cables that crossed the holdfast and enclosed the entire line of cultures, which remained in the culture system for 60 days (for more details, see [26]).

After the specified time, the 15 kelps cultured in the HIZ were removed and translated (in the same way as mentioned above) to CIMARQ to be maintained in tanks (covered 5000-L Raceway-type tanks with an open flow of seawater filtered at 1 µm and constant aeration) for subsequent use in the feeding experiments. Additionally, a second group of 15 kelps was directly collected from Algarrobo (NIZ group), which were also kept in tanks at CIMARQ. From these two groups, blade samples of 250 g were collected in triplicate, stored in aluminum foil packages, and frozen at −20 °C for the subsequent analysis of PAH and HM concentrations.

### 2.1. Feeding Experiment

Twenty-four adult individuals of *T. niger* (with a head diameter of 85–100 mm) were manually collected from Caleta Quintay (33°11′36″ S, 71°41′59″ W). These individuals were acclimatized for 2 days in a fasting state in 15-L glass tanks containing seawater filtered at 1 µm, with open flow and constant aeration, a temperature between 15 and 17 °C, and natural light. These 24 individuals were then assigned at random to the NIZ or HIZ treatments, consisting of 4 tanks per treatment with 3 sea urchins each, and these tanks are considered to be replicates (Figure 2). Specifically, the sea urchins were fed 200 g of fresh algae per tank from the NIZ or HIZ. The experiment lasted 3 weeks. Every week, the tanks were cleaned, and the sea urchins were given 200 g of additional fresh algae after removing the algae from the previous week. 

### 2.2. Analysis of PAHs in Algal and Animal Tissues

After the feeding experiment, the gonads were removed, corresponding to 1.5 kg of sea urchins per treatment, including the control group. The gonads were stored in aluminum foil packages and frozen at −20 °C for subsequent analysis. The gonads were extracted after inducing the release of gametes. The tissues of the above mentioned algae from both NIZ and HIZ were analyzed in the Análisis Ambientales S.A. (ANAM) laboratory (https://www.anam.cl, accessed on 1 September 2021), which performed assays for 16 PAH-type compounds: acenaphthene, acenaphthylene, anthracene, benzo[a]anthracene, benzo[a]pyrene, benzo[b]fluoranthene, benzo[ghi]perylene, benzo[k]fluoranthene, chrysene, dibenzo(ah)anthracene, phenanthrene, fluoranthene, fluorene, indeno[1,2,3-cd]pyrene, naphthalene (NAF), and pyrene. These PAHs correspond to the 16 compounds listed by the United States Environmental Protection Agency (US EPA) as high priority due to their toxicity in animal organisms and their persistence in the environment. The algal tissue was treated as sludge; therefore, the assays were performed as if they were marine sediment. The sea urchin gonads were analyzed at the Measurement Studies and Quality Certification Center (CESMEC S.A.) of the Bureau Veritas Group (https://www.cesmec.cl, accessed on 1 September 2021), and assays were performed for the same compounds as those analyzed in algal tissue. Both species were analyzed using modified 6440B and 6410B methods in the Standard Methods for Examination of Water and Wastewater, 23rd edition (American Public Health Association, American Water Works Association, Water Environment Federation ©2018, available at https://standardmethods.org, accessed on 1 September 2021). 

The tissue samples (sea urchin gonads and algae) were homogenized and mixed with dichloromethane to extract PAHs. After extraction, the solution was filtered, and the supernatant was concentrated in a Kuderna–Danish (K-D) concentrator at 60–65 °C until the solvent had almost completely evaporated. The extract was recovered with 4 mL of cyclohexane in an amber vial. A cleanup step was performed to remove impurities and interfering substances. The extract was eluted through a 10 mm ID glass column with 10 g of activated silica gel and 1–2 cm of anhydrous Na_2_SO_4_ on the silica. Pentane (40 mL) was then added to the column, which was discarded and then the extract was eluted with 25 mL of dichloromethane/pentane (4:6 v/v). The extract was concentrated with a K-D concentrator to a final volume of 1 mL. Naphtalene-d_8_ and benzo[a]anthracene were used as internal standards.

Final determination was carried out by a gas chromatograph/mass spectrometer (GC/MS) using Supelcoport (100/120 mesh) coated with 1% SP-1240DA packed in 1.8 m long, 32-mm ID glass columns with helium carrier gas at a 30 mL/min flow rate. Column temperature was held isothermal at 70 °C for 2 min, then programmed to increase to 200 °C at a rate of 8 °C/min. A 1-μL sample was injected into the column using splitless mode. The injector temperature was 250 °C. The mass spectrometer was operated in a selected ion monitoring (SIM) mode with an electron impact ionization of 70 eV.

### 2.3. Sampling and Analysis for Heavy Metals

Three 500 g samples of *M. pyrifera* and three 500 g samples of adult *T. niger* individuals were collected directly (not subjected to a feeding trial step) from natural populations of Algarrobo (NIZ) and Caleta Horcón (HIZ). The specimens were packaged in clean plastic bags, transported in cold containers (5–8 °C), and kept frozen prior to analysis. Both the *M. pyrifera* and *T. niger* samples were analyzed at CESMEC by atomic absorption after acid digestion. For each matrix, analyses were performed to measure As, Cd, Cu, and Pb, which have been reported to negatively impact the development of sea urchin larvae and on the macroalgae populations in areas surrounding the Ventanas industrial park [23,50,51,52,53]. Each metal was analyzed using the corresponding methodology according to the Chilean National Institute of Standard-Setting (INN), available online at https://www.cesmec.cl/medios/documentos/AcreditacionesINN/ensayos/LE080.pdf (accessed on 1 May 2021). 

### 2.4. Ecotoxicological Indices and Factors

For HMs, the following pollution indices were calculated: the metal pollution index (*MPI*), the pollution load index (*PLI*), the bioconcentration factor (*BCF*), and the trophic transfer factor (*TTF*). For both the *MPI* and *PLI* in the water column, the metal concentration data sets used were those reported by the General Directorate of Maritime Territory and Marine Merchants (DIRECTEMAR, https://www.directemar.cl, accessed on 1 September 2021) and [23]. 

To compare the total content of metals in the different zones and biological matrices, we calculated the *MPI*, where the following formula was used according to [54]:

(1) $$MPI=\sqrt[{n}]{Cf_{1}*Cf_{2}*\ldots *Cf_{n}}$$

where *Cf* is the concentration of metal *n* in the sample.

The *PLI* was calculated and used to compare the pollution status of the sites. This index is used to demonstrate the availability of metals in the water column and, therefore, the status of coastal health. The following formula was used according to [55]:

(2) $$PLI=\sqrt[{n}]{CF_{1}*CF_{2}*\ldots *Cf_{n}}$$

(3) $$CF=C_{metal}/C_{baseline}$$

where *CF* is the contamination factor of metal *n*, which is calculated as the ratio of the measured concentration of the metal in the sample (*C_metal_*) to the background value (or *C_baseline_*); *C_baseline_* is the lowest concentration of the metal reported in the study or any quality parameter, which in this case was the US EPA standard value for the metal (https://www.epa.gov/wqc/national-recommended-water-quality-criteria-aquatic-life-criteria-table#table, accessed on 1 September 2021). *PLI* > 1 denotes a polluted condition, whereas *PLI* < 1 indicates no metal pollution [56].

To obtain the bioconcentration factor (*BCF*) of algal tissue pollutants, the PAH and HM concentrations in the water column published by DIRECTEMAR were used for both the NIZ and HIZ, except for the HMs in the HIZ, for which the concentrations reported in [23] were used. The *BCF* was obtained using the following formula according to [57]:

(4) $$BCF=\frac{C_{a}}{C_{w}}$$

where *C_a_* is the measured concentration of the compound in *M. pyrifera* tissue, and *C_w_* is the concentration of the compound in the water column. 

The trophic transfer factor (*TTF*) was obtained using the following formula according to [58]:

(5) $$TTF=\frac{C_{e}}{C_{a}}$$

where *C_e_* is the measured concentration of the compound in *T. niger* gonads, and *C_a_* is the measured concentration of the compound in *M. pyrifera* tissue.

Depending on the values obtained for both factors (i.e., *BCF* and *TTF*) under the given conditions, the organisms can be characterized as hyperaccumulators (values over 1) or as capable of excluding pollutants (values under 1) [57,58].

### 2.5. Statistical Analysis

To identify statistically significant differences in the PAH and HM concentrations between the treatments and biological matrices, a one-way analysis of variance (ANOVA) was performed with a significance of 95%, that is, *p* < 0.05. Prior to this, descriptive analyses were performed to evaluate the normality and homogeneity of variance. Three replicates were performed for both the algal tissue and sea urchin gonads. All the analyses were performed in R [59]. For both the toxicity indices and factors, statistical analyses were not performed; rather, the ratios between the treatments and matrices were compared.

## 3. Results

**Figure 3:**
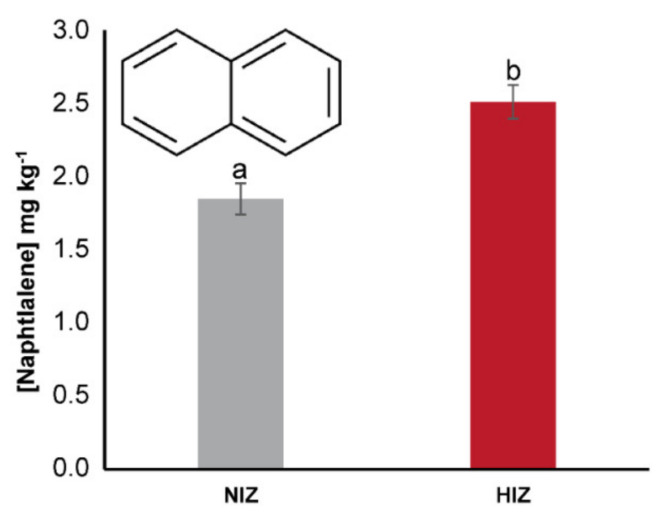
Concentration of naphthalene (NAF) in *Macrocystis pyrifera* tissue from Algarrobo (NIZ) and Caleta Horcón (HIZ) after 60 days of culture. Letters (a and b) correspond to significant differences, ANOVA (*p* < 0.05). Each treatment comprised three replicates. NIZ = non-impacted zone, HIZ = highly impacted zone.

In *Macrocystis pyrifera* tissues, the concentrations of most analyzed PAHs were under the detection limit, except for naphthalene (NAF), whose concentrations were above its detection limit (0.08 mg kg^−1^) and were 1.8 mg kg^−1^ for algae from the non-impacted site (Algarrobo, NIZ) and 2.5 mg kg^−1^ for algae from the highly impacted site (Caleta Horcón, HIZ) (Figure 3). The concentrations reported by DIRECTEMAR for NAF bordered on the detection limits for the water column at both sites, although in the most recent annual report for the study period, a NAF concentration of 5 × 10^−5^ mg L^−1^ was recorded. For the PAH concentrations in the *T. niger* gonads, all values were below their detection limits.

The NAF *BCF* of *M. pyrifera* was 3693 for NIZ and 5020 for HIZ. Since the PAH concentrations in the *T. niger* gonad tissue were below the detection limit, the *TTF* was not applicable to this sea urchin in this study.

**Figure 4:**
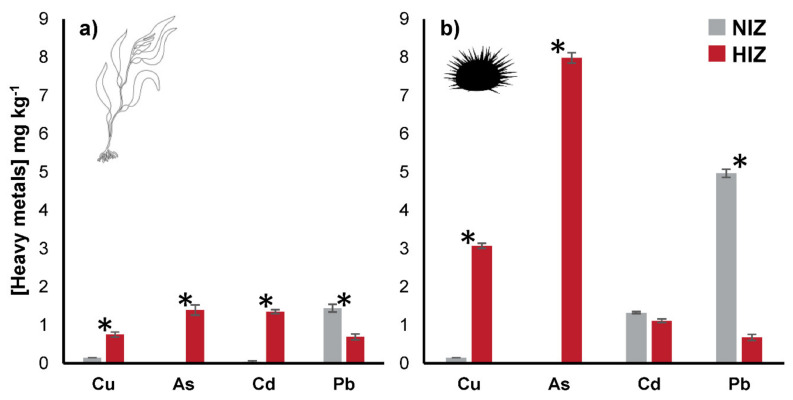
Concentrations of Cu, As, Cd, and Pb in (**a**) *M. pyrifera* and (**b**) *T. niger* tissues collected from Algarrobo (NIZ) and Caleta Horcón (HIZ). Asterisks represent significant differences between the treatments of the same matrix for each metal, ANOVA (*p* < 0.05). NIZ = non-impacted zone, HIZ = highly impacted zone.

In both the *M. pyrifera* tissue and the *T. niger* gonads, the concentrations of HMs were higher in the HIZ than in the NIZ treatment (Figure 4). Regarding Cu concentration in the HIZ samples, the measured concentrations were 0.75 mg kg^−1^ in the algal tissue and 3.07 mg kg^−1^ in the sea urchin gonads. For As, the measured concentrations were 1.39 and 7.98 mg kg^−1^ in the algae and sea urchins, respectively, from the same site. However, the concentrations of Cu and As in the samples from the NIZ were under the detection limit (Figure 4). Finally, the concentrations of Cd in the algal tissue were 0.04 mg kg^−1^ in the NIZ and 1.35 mg kg^−1^ in the HIZ, and the difference was statistically significant (in all cases, *p* < 0.05). In *T. niger* gonads, the Cd concentrations were 1.32 and 1.11 mg kg^−1^ for the NIZ and HIZ, respectively, and were not significantly different (Figure 4).

Notably, the pattern of Pb concentrations in both sites differed from those of the previously mentioned metals. Concentrations of 1.44 and 4.96 mg kg^−1^ were recorded for algae and sea urchins from the NIZ, and 0.69 and 0.68 mg kg^−1^ were measured for the HIZ, respectively, with no statistically significant differences identified between the biological matrices (*p* > 0.05; Figure 4).

With respect to the pollution indices, the values for *MPI* were higher for the HIZ samples than the NIZ samples for all three matrices. In both zones, the metal contamination increased with the increasing ecosystem matrix level and/or higher trophic level; for example, in the HIZ, the *MPI* values were 80.4 × 10^−4^ in the water column, 0.99 in *M. pyrifera* tissue, and 2.07 in *T. niger* gonads (Table 1). According to the *PLI*, Caleta Horcón is a highly polluted site based on the US EPA standards, as it had a value much higher than 1 (10.896), while Algarrobo, with an index value lower than 1 (0.015), can be characterized as a relatively non-polluted site (Table 1).

As opposed to the PAH analyses, the *BCF* and *TTF* of metals could be determined. However, these factors could not be calculated in the NIZ for Cu and As since their concentrations were under the detection limit. For both factors, higher values were obtained in the HIZ than in the NIZ, except for Pb, where the values in the NIZ were higher for both *M. pyrifera* and *T. niger* (1152 *BCF* and 3.4 *TTF* vs. 475.3 *BCF* and 1.0 *TTF*, respectively; Table 2). In particular, the *BCF* in the HIZ fluctuated between 3.4 and 4214, reaching the highest bioconcentration for Cd in *M. pyrifera*. The largest transfer recorded to *T. niger* was for As (5.7 *TTF*; Table 2).

## 4. Discussion

According to the results obtained in this study, specifically for *MPI* and *PLI*, Caleta Horcón (HIZ) has a greater load of pollutants than Algarrobo (NIZ), mainly with respect to heavy metals, which surpass the regulations established by the US EPA. This polluted condition is similar to those reported by other studies, such as [60], who studied the concentrations of different metals in the Gulf of Chabahar, where a landing port is located. In their work, the *PLI* (using the same US EPA standard mentioned above) and *MPI* in the water column were 30.09 and 0.026, respectively, which result in this area being characterized as a highly impacted zone. Other similar cases are the Bay of Jinzhou, China [31], and the Port of Canakkale, Turkey [61]. Thus, Caleta Horcón can be classified as a zone of high industrial impact due to the measured concentrations of heavy metals and NAF in *M. pyrifera*, and because it has a significantly higher load than Algarrobo. In this context, the differences in the impact of the zones are discussed below. 

### 4.1. BCF and Transfer of PAHs in M. pyrifera and T. niger

As previously mentioned, reports on the level of pollution at sites located near the highly industrialized area of Quintero Bay have identified the presence of several PAHs that are found at relatively high concentration in the seawater (6.23–17.61 µg L^−1^ [25]). As such, given the absence of other PAHs in the tissue of *Macrocystis pyrifera*, the high concentrations of naphthalene (NAF) alone are probably not indicative of the exclusive presence of NAF in the seawater. The high *BCF* of NAF by *M. pyrifera* for both the NIZ and HIZ confirmed this trend of high NAF accumulation in this species. The NAF levels in *M. pyrifera* in this study were higher than those previously reported [62] in the brown algae *Padina boryana*. In that study, the same compounds were analyzed in algal tissue, and whereas the concentrations of the other PAHs were above the detection limit, the concentrations of NAF in *M. pyrifera* were 3.6 and 5.1 times higher in NIZ and HIZ, respectively, than those found in *P. boryana*. Algarrobo (NIZ) shows no indication of an environmental impact due to pollutants from industrial activities, but the high level of NAF in *M. pyrifera* suggests that some pollutants may come from domestic wastes and port activities, primarily associated with tourism and recreational activities. Conversely, Caleta Horcón (HIZ), which is located near an industrialized park, receives industrial pollutants directly from aquatic and air sources, as well as oil spills. This could largely explain the higher concentration of NAF in the HIZ compared with the NIZ. Nonetheless, the high concentration of NAF found in *M. pyrifera* tissue from Algarrobo (NIZ) in this study also indicates that further research is needed to better understand the sources of pollution in this zone, which was considered, a priori, a relatively unpolluted area. 

The absence of other PAHs in both analyzed biological matrices could be due to biodegradation mechanisms or to the small size of NAF compared to other PAHs. As previously established [63], small molecules such as NAF can permeate phospholipid membranes, with diverse naphthalene derivatives exhibiting different preferences for the hydrophilic or hydrophobic domain of phospholipids. This mechanism may explain the high NAF levels observed in *M. pyrifera*. Conversely, just as some hydrocarbons can be synthesized by marine organisms, PAHs can also be degraded, as was determined in *Chlorella sorokiniana* and *Ulva fasciata* [64]. This process is the result of enzymatic activity in the presence of molecular oxygen, which catabolizes the benzene rings that are characteristic of these compounds [65,66]. In a study performed in 1998 where *Fucus vesiculosus* was used to determine the destination of benzo[a]pyrene, Kirso and Irha [67] demonstrated that after 10 days of exposure, about 96% of the pollutant was found in the algal tissue, and up to 4% could not be found in the aquatic matrix or in the algal tissue. The authors suggested that this portion was metabolized by the algae, as described in other studies [68,69]. However, the lighter PAHs (NAF, for example) should biodegrade more quickly than the heavier ones [70]. The high levels of NAF in algal tissue may be because *M. pyrifera* does not have a similar degradation mechanism to that described in other species. In addition, the results of this study do not provide evidence of the transfer of NAF through the food chain (that is, through *M. pyrifera*) to sea urchin because of the low concentrations of this compound in *T. niger* gonads. For the sea urchin *T. niger*, degradation mechanisms could be mainly influencing this absence of NAF and other PAHs in its tissue. However, again, this does not mean that these pollutants are not being ingested by the herbivore, as they may also be metabolized. It was reported that both fish and other marine organisms can metabolize PAHs through a group of enzymes dedicated to the oxygenation of these compounds and that the cytochrome P-450 monooxygenases (P450s) of fishes are able to oxygenate PAHs [71]. When the crab *Sylla serrata* was directly exposed to sublethal concentrations of NAF, its enzymatic activity in the hemolymph was found to increase, which indicates that its mechanisms of tolerance to NAF occur through its metabolization [72]. Nonetheless, the detection limit for NAF (0.08 mg kg^−1^) in this study was relatively high. Further research in *T. niger* must be conducted using an analytical procedure allowing for the detection of a lower concentration of PAHs, i.e., a more sensitive method with a lower detection limit, below the maximum concentrations of PAHs recommended by international criteria for marine herbivores. Alternatively, assuming an absence of PAHs in *T. niger* tissue, another possible explanation is that the PAHs are mainly bioconcentrated in the gonads [73,74], and because our measurements were performed after the release of gametes, their concentrations could have dropped to below the detection limit. However, more information is required to determine the possible events or mechanisms that explain the low detected levels of NAF in *T. niger* after the consumption of *M. pyrifera* with high levels of this PAH. Although the negative effects of PAHs have been demonstrated in sea urchins, few studies have examined the bioaccumulation and dynamics of pollutant transfer from their diet [75,76]. This is important because the bioaccumulation of PAHs may affect the persistence of herbivores consuming polluted algae and have indirect effects on their predators; for example, PAHs such as fluoranthene, pyrene, and phenanthrene were found to affect the axial development of sea urchin embryos by interrupting the regulation of β-catenin, which is an important transcriptional regulator in embryo development [77,78,79]. It is worth mentioning that due to the physicochemical properties of PAHs, they can precipitate and remain in the solid fraction according to their structural differences [80,81]; then, an analysis of the marine sediments must be considered in future studies. In our case, for most PAHs the concentration measured in the alga and the sea urchin were below the instrumental detection limit, which could have resulted from the precipitation of these compounds in the marine sediments.

### 4.2. BCF and Transfer of Heavy Metals in M. pyrifera and T. niger 

Similar to PAHs, heavy metals (HMs) are also introduced in large quantities and mainly originate from anthropogenic sources associated with both industrial and domestic activities. One study [37] demonstrated that the concentrations of HMs in *M. pyrifera* are closely related to their concentrations in the water column. This is consistent with the results obtained in our study, where *BCF* > 1 was determined for *M. pyrifera* in the HIZ for all metals and was higher than that in the NIZ, thereby forming the basis for classifying *M. pyrifera* as a hyperaccumulating species with respect to metals. Additionally, heavy metal concentrations in the seawater and sediment have risen considerably in the HIZ during the last two decades, probably extending its pollution load to northern coastal sites according to Oyarzo-Miranda 2020 [23]. As in the case of NAF in *M. pyrifera*, the *BCF* for Cd and Pb in *T. niger* in the NIZ was also high in our study, suggesting that an important pollution load occurs in this zone. In this sense, to better understand this phenomenon, it is necessary to conduct further research on the combined effects and the dynamic uptake of these metals when they co-occur.

Some metals, such as Pb, are not only derived from industrial activity but also domestic and transport sources, and the main form of entry of Pb into the water is through the atmosphere as particulate matter [82]. This means that the distribution of Pb along the coastline is ubiquitous, presenting focal points of higher pollution in the presence of human settlements [82,83], which also explains the important pollution load observed in the NIZ both for NAF in *M. pyrifera* and Pb in *T. niger*. This context and the low amount of Cu and Cd detected in the NIZ water column suggest that the low Pb concentrations in algal and *T. niger* tissues from Caleta Horcón (HIZ) are due to the antagonistic dynamics of the cellular incorporation of this metal, as previously demonstrated [84,85]. This is further evidence of the chemical interaction between toxic elements and their possible effects on marine organisms. 

The heavy metals Cu, As, and Cd are characteristic pollutants in industrial zones, primarily resulting from mining activity and the burning of fossil fuels. Through metabolic mechanisms, algae can actively bioconcentrate metals at the intracellular level, but initially, rapid and passive adsorption occurs on their surfaces [86]. During the active phase, metal ions are transported through and toward the cytoplasm, a process which may accelerate upon combination with intracellular compounds (such as glutathione) [35,87], thus accumulating within the cell. The excess bioaccumulation of heavy metals in algae, as previously mentioned, induces the rapid accumulation of ROS, generating oxidative stress. The accumulation of ROS above the upper limit of tolerance in the algal species may, in turn, damage macromolecules and cell structures [20,21], and the heavy metals may be transferred to herbivores, such as *T. niger*, through the food web. As a result, heavy metals generate direct and indirect effects on higher organisms in the food web. Nonetheless, brown algae such as *Dictyota kuntii* possess diverse mechanisms to counteract metal excess, such as metal accumulation, the activation of antioxidant enzymes, and inducing the release of metal-binding compounds in their exudates [88]. Such mechanism may allow *M. pyrifera* to tolerate the high levels of metals occurring in the seawater of the HIZ, assuming the existence of similar tolerance mechanisms in this species to *Dictyota kuntii*. 

*T. niger* is characterized as an herbivore that feeds on macroalgae, mainly kelps, including *M. pyrifera* (mainly washed ashore) and *Lessonia berteroana* [45]. Given this feeding behavior, and according to the *TTF* of *T. niger* determined in this work, it can be inferred that a large quantity of the pollutants recorded in this herbivore were from its diet. The concentrations of metals in sea urchin gonads increase during the feeding stage because of the greater ingestion of pollutants through its food. The individuals from Algarrobo (NIZ) had higher concentrations of Cd and Pb, which are transferred more easily through the food web in the absence of other metals. The individuals from Caleta Horcón (HIZ) had *TTF* ≥ 1, except for Cd. An explanation for this may be that the distribution of HMs is heterogeneous throughout the sea urchin body [89], and Cd tends to accumulate in hard structures such as the shell [90,91]. Therefore, compared to other pollutants (e.g., As and Cu) that tend to accumulate in the soft tissue [91], the Cd transferred from *M. pyrifera* does not necessarily accumulate in *T. niger* gonads. Alternatively, the high accumulation of Cu compared to the relatively low accumulation of Cd in both *M. pyrifera* and *T. niger* could be explained by the competitive uptake of these two metals in favor of Cu, such as in the microalga *Chlorella* sp. or the kelp *L. berteroana*, where cadmium and copper co-exposure increases copper uptake levels but inhibits those of cadmium compared to when each metal is present alone [92,93]. 

The results obtained in this research reflect not only an environmental but also a socio-environmental problem. The concentrations of heavy metals found in both the algal tissue and the gonads exceed the limits stipulated in Codex Alimentarius (Norm 193-1995, FAO) for human and animal consumption. In addition, the International Agency for Research on Cancer (IARC) assigned naphthalene to Group 2B—possibly carcinogenic to humans—because there is sufficient evidence for the carcinogenicity of this compound in experimental animals. Therefore, in organisms with eating behaviors similar to those of *T. niger*, such as the red sea urchin *Loxechinus albus* [94,95], a species of commercial importance and consumed as human food, the presence of these contaminants could generate negative effects on human health. However, any conclusion regarding this requires further analysis, though should be considered with caution.

Finally, it is worth mentioning that for both the calculation of the *MPI* and *PLI*, the data sets used regarding metals concentrations in the water column were those reported by previous studies. This could have affected the magnitude of these pollution indices and of the interpretation of the bioconcentration of the pollutants in *M. pyrifera* and *T. niger* tissues, especially considering that the pollution load in this zone is probably highly variable (see, for example, the ranges of HMs concentrations reported by [23]) depending on the discrete events of shedding of pollutants by industries or port activities or the re-suspension of HMs or PAHs by ocean currents. Then, future studies in this zone will have to consider a synchronous measurement of HMs or PAHs both in the water column and in these two types of organisms to more strongly confirm the pollution patterns observed. Nonetheless, neither the clear difference of the concentrations nor ecotoxicological indexes patterns of toxics between HIZ an NIZ should change in future studies. On the other hand, as pointed out by studies such as [96], heavy metals can be transported by airborne particles, which can be driven far away from their sites of emission. Accordingly, it would also be useful to investigate the heavy metals content in airborne particles from the Quintero Bay industrial and surrounding areas, to better define the sources of pollutants in the HIZ and the NIZ.

## 5. Conclusions

The results obtained in this study showed that Caleta Horcón (HIZ) is a more polluted marine zone than Algarrobo (NIZ). This difference in the level of pollution stems mainly from the higher concentrations of heavy metals and NAF in the seawater and biological matrices of the HIZ than NIZ. Nonetheless, as previously mentioned, the high bioaccumulation of Cd (1.32 mg kg^−1^) and Pb (0.69 mg kg^−1^) in *T. niger* and NAF in *M. pyrifera* (1.8 mg kg^−1^) within the NIZ suggest the existence of a relatively high pollution load also in this zone; however, further research is needed to more accurately determine which are the sources of these pollutants. In general, an order of increasing metal concentrations was verified in seawater, *M. pyrifera*, and *T. niger*, especially in the HIZ; conversely, for NAF, this trend was only verified in *M. pyrifera* but not *T. niger*. In the case of HMs, we can infer that HMs transfer via the diet occurred because *T. niger* had higher concentrations of metals than *M. pyrifera*, with *TTF* > 1 for most metals at both sites, which is in accordance with the feeding behavior of *T. niger*. The former findings are important because these pollutants may have direct and indirect harmful effects on algae and their herbivores, as demonstrated previously for early developmental stages and adult individuals of kelps and sea urchin. 

## Figures and Tables

**Table 1:** Metal pollution index (*MPI*) and pollution load index (PLI) values determined in the water column, algal tissue, and sea urchin gonads of Algarrobo (NIZ) and Caleta Horcón (HIZ), as applicable. NIZ = non-impacted zone, HIZ = highly impacted zone.

Index	Treatment	Water Column	*M. pyrifera* Tissue	*T. niger* Gonad
*MPI*	NIZ	6.6 × 10^−4^	0.49	1.60
	HIZ	80.4 × 10^−4^	0.99	2.07
*PLI*	NIZ	0.015		
	HIZ	10.896		

**Table 2:** *BCF* and *TTF* for Cu, As, Cd, and Pb in *Macrocystis pyrifera*/water column and *T. niger/M. pyrifera*. N.D. = the concentration of the metal is below the limit of detection, NIZ = non-impacted zone, and HIZ = highly impacted zone.

Factor	Treatment	Species	Cu	As	Cd	Pb
*BCF*	NIZ	*M. pyrifera*	N.D.	N.D.	80.0	1152
	HIZ		34.2	3.4	4214	475.3
*TTF*	NIZ	*T. niger*	N.D.	N.D.	33.0	3.4
	HIZ		4.1	5.7	0.8	1.0

## Data Availability

Derived data supporting the findings of this study are available from the corresponding author (L. Contreras-Porcia) on request.
